# The transporter GAT1 plays an important role in GABA-mediated carbon-nitrogen interactions in *Arabidopsis*

**DOI:** 10.3389/fpls.2015.00785

**Published:** 2015-09-29

**Authors:** Albert Batushansky, Menny Kirma, Nicole Grillich, Phuong A. Pham, Doris Rentsch, Gad Galili, Alisdair R. Fernie, Aaron Fait

**Affiliations:** ^1^The Albert Katz International School for Desert Studies, The Jacob Blaustein Institutes for Desert Research, Ben-Gurion University of the Negev, Midreshet Ben-GurionBeer Sheva, Israel; ^2^Department of Plant Science, Weizmann Institute of ScienceRehovot, Israel; ^3^Central Metabolism Group, Willmitzer Department, Max-Planck Institute of Molecular Plant PhysiologyPotsdam-Golm, Germany; ^4^Department of Biology, Institute of Plant Sciences, University of BernBern, Switzerland

**Keywords:** GABA, glutamate, carbon nitrogen metabolism, GAT1, GC-MS profiling

## Abstract

Glutamate derived γ-aminobutyric acid (GABA) is synthetized in the cytosol prior to delivery to the mitochondria where it is catabolized via the TCA cycle. GABA accumulates under various environmental conditions, but an increasing number of studies show its involvement at the crossroad between C and N metabolism. To assess the role of GABA in modulating cellular metabolism, we exposed seedlings of *A. thaliana* GABA transporter *gat1* mutant to full nutrition medium and media deficient in C and N combined with feeding of different concentrations (0.5 and 1 mM) of exogenous GABA. GC-MS based metabolite profiling showed an expected effect of medium composition on the seedlings metabolism of mutant and wild type alike. That being said, a significant interaction between GAT1 deficiency and medium composition was determined with respect to magnitude of change in relative amino acid levels. The effect of exogenous GABA treatment on metabolism was contingent on both the medium and the genotype, leading for instance to a drop in asparagine under full nutrition and low C conditions and glucose under all tested media, but not to changes in GABA content. We additionally assessed the effect of GAT1 deficiency on the expression of glutamate metabolism related genes and genes involved in abiotic stress responses. These results suggest a role for GAT1 in GABA-mediated metabolic alterations in the context of the C-N equilibrium of plant cells.

## Introduction

γ-Aminobutyric acid (GABA) was first extracted from plants in the middle of the previous century (Steward et al., 1949). Due to its intermediate position between glutamate and the TCA cycle, the GABA shunt is intimately connected to the C-N balance and partitioning of the plant and a central role of GABA metabolism at the junction of carbon (C) and nitrogen (N) metabolism was recently emphasized (Fait et al., 2008, 2011). Studies have shown that the key regulatory enzyme of GABA biosynthesis is glutamate decarboxylase (GAD) whose overexpression in transgenic plants significantly reduced the levels of glutamate (Baum et al., 1996; Bouché and Fromm, 2004). This observation suggests that GABA could participate in the regulation of N metabolism and uptake (Forde, 2002) yet raised questions concerning the transport of GABA both within the plant and within the plant cell. That being said and despite the fact that the biosynthesis and metabolism of this compound are relatively well-characterized (Baum et al., 1993; Shelp et al., 1999), the transport of GABA is less well-researched.

Evidence of the enhanced growth of *Arabidopsis* supplied with GABA previously suggested the existence of GABA transporters, confirmed by the identification of proline transporter 2 (ProT2) as a quaternary transporter which mediates the influx of both GABA and proline with preference to the latter substrate (Breitkreuz et al., 1999). The first GABA specific transporter in plants, GAT1, was isolated from the cell membrane of *Arabidopsis thaliana* (Meyer et al., 2006). In contrast to ProT2 GAT1 does not transport proline but shows high affinity to GABA compared to other substrates such as alanine and β-alanine (Meyer et al., 2006). Only recently was the transport of GABA to the mitochondria also, at least, partially elucidated by the isolation of a GABA-permease (GABP), localized to the mitochondrial membrane (Michaeli et al., 2011). In spite of these recent findings the functional role of GABA transport and its involvement in modulation of C-N metabolism remains elusive. Here we studied the metabolism and gene expression of the *Arabidopsis* mutant *gat1*, lacking the GABA transporter GAT1, under different C-N media and when supplemented with exogenous GABA.

## Materials and methods

### Chemicals

All chemicals were purchased from Sigma-Aldrich Israel Ltd. (Jerusalem, Israel) with the exception of *N*-methyl-*N*-[trimethylsilyl]-trifluoroacetamide (Macherey-Nagel GmbH & Co. KG, Düren, Germany).

### Plant material and growing conditions

*Arabidopsis thaliana* ecotype *Wassilewskija gat1* lines were isolated from pools of the lines created and published by Patrick J. Krysan and coauthors (Krysan et al., 1996) using the gene specific primer (T23G18-R3) 5′-GATGGGAATTGTGCCAAAACC-3′ and T-DNA primer (F-LB) 5′-GATGCACTCGAAATCAGCCAATTTTAGAC-3′. The mutation was validated by the results of RT-PCR that did not reveal any AtGAT1 mRNA in the *ws* gat1 T-DNA insertion line, while after the same number of cycles in wild type plants the transcript were amplified. The seeds of the mutants lacking GAT1 in the *ws* background were sterilized with 50% commercial bleach for 5 min, and then three times washed with distilled water. After sterilization seeds were sown in the semiliquid sterile full nutrition (FN) media consisting of 2 mM KNO_3_, 1 mM NH_4_NO_3_, 1 mM Gln, 3 mM KH_2_PO_4_/K_2_HPO_4_, pH 5.8, 4 mM CaCl_2_, 1 mM MgSO_4_, 2 mM K_2_SO_4_, 3 mM MES, pH 5.8, 0.5% (w/v) sucrose, and microelements (i.e., 40 mM Na_2_FeEDTA, 60 mM H_3_BO_3_, 14 mM MnSO_4_, 1 mM ZnSO_4_, 0.6 mM CuSO_4_, 0.4 mM NiCl_2_, 0.3 mM HMoO_4_, 20 mM CoCl_2_). The seedling were incubated in 25 ml of FN sterile liquid cultures (250 ml Erlenmeyer glass flasks) on orbital shakers with constant speed, uniform fluorescent light (±50 μE in the flask) and temperature (21 ± 1°C) at 18:6 h (L:D) for 14 days. Thereafter the seedlings were transferred to new flasks with different freshly prepared media: FN, C, and N deficiency (12 flasks per media) for additional 24 h. The medium for C deficiency had the same composition as FN but without 0.5% (w/v) sucrose; the medium for N deficiency contained 0.1 mM KNO_3_, 50 μM NH_4_NO_3_, no Gln, and 3 mM KCl, and all other components as in FN media (Scheible et al., 2004; Osuna et al., 2007). Then, GABA at 0.5 and 1 mM concentrations was added to each medium (four flasks per concentration). Following 6 h after GABA addition seedlings from each flask were collected, damped, separately homogenized in liquid nitrogen and kept at −80°C until further analysis.

### Metabolite extraction and analysis

Metabolite extraction from 100 mg of *Arabidopsis* seedlings followed an established protocol (Lisec et al., 2006). 100 μl of the extract were dried in a vacuum concentrator (Eppendorf Concentrator Plus). Ahead of injection, samples were derivatized by adding 40 μl of fresh solution of methoxyaminhydrochloride in pyridine (20 mg ml^−1^); (Lisec et al., 2006). Following derivatization, samples were transferred to GC-MS auto-sampler glass vials. Chromatographic separation was carried out on a Thermo Scientific DSQ II GC/MS using a FactorFour Capillary VF-5 ms column.

### Metabolite annotation and data analysis

Chromatograms were analyzed using TagFinder04 software 1.0 (Luedemann et al., 2008). The mass spectra were compared to an available standard library from Golm database (Hummel et al., 2007). Principal component analysis (PCA) and tests of significance were performed on the data sets obtained from metabolite profiling with the software package tMEV (Saeed et al., 2003). Prior to the analysis, data were normalized to the exact weight of each sample and internal standard (ribitol) and Log-transformed. Pairwise Student's *t*-test with confidence interval 95% was used to compare two genotypes within each media. Differences between treatments were tested for significance by One-Way ANOVA with confidence interval 95% and Bonferroni correction.

### Total RNA extraction and gene expression analysis

Based on the results of metabolite analysis, RNA extraction was carried out on seedlings from the different treatments with and without 1 mM GABA only (skipping intermediate GABA concentration), using the standard TRIzol (Invitrogen) protocol. Whole transcriptome analysis was performed using microarray technology according to the standard Affymetrix protocols (available online http://www.affymetrix.com/support/technical/manuals.affx) on *Arabidopsis* Genome ATH1 Array chip. Each test was performed on two biological replicates. Briefly, on the first stage first-strand cDNA was synthesized from RNA sample and, then was converted into a double stranded cDNA (dsDNA). The reaction employs DNA polymerase and RNase to simultaneously degrade the RNA and synthesize second-strand cDNA. Labeling, hybridization, scanning, and data extraction were performed by Affymetrix protocols. Analysis of transcriptomic data was done using Partek Genome Suite software (Partek); (www.partek.com). Pre-processing was carried out using the Robust Microarray Averaging (RMA) algorithm (Irizarry et al., 2003) and then Two-Way ANOVA was performed. False discovery rate (FDR) method was used to decrease the false-effect of multiple comparisons. Differentially expressed genes were chosen according to *p*_*FDR*_ < 0.05 and ≥ 4 times fold-change. Functional annotation, overrepresentation analysis and metabolic pathways annotation was performed by The Database for Annotation, Visualization and Integrated Discovery (DAVID) resource (http://david.abcc.ncifcrf.gov).

## Results

**Figure 1:**
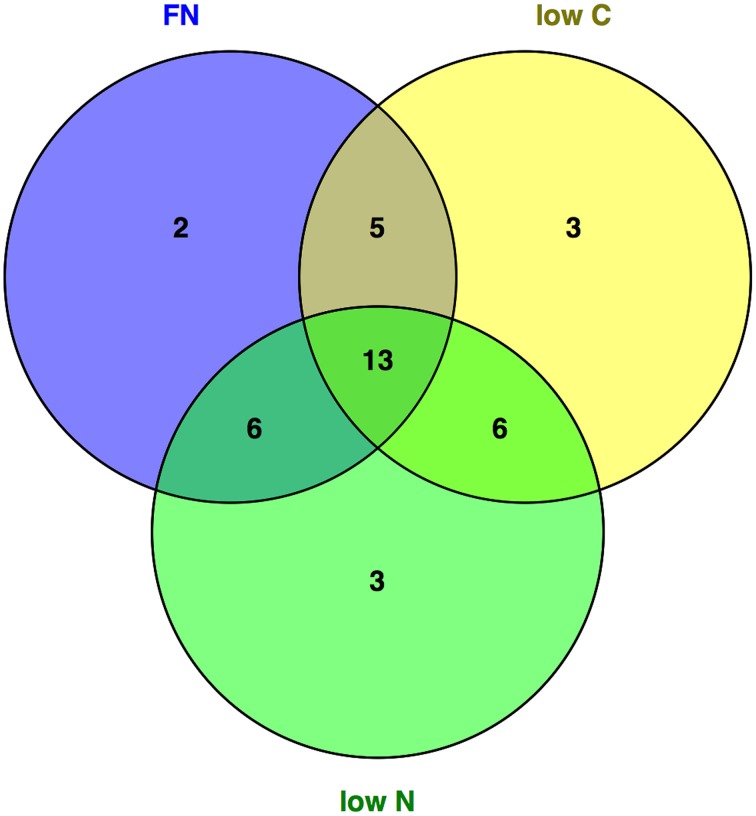
**General and medium-specific metabolite response of *gat1* compared to *ws***. Metabolites that changed significantly (*p* < 0.05) in *gat1* compared to *ws* across media were plotted using a Venn Diagram to identify communal and medium specific response.

The analysis of central metabolites by GC-MS showed that of the 44 uniquely and unequivocally annotated metabolites 59, 61, and 63% changed significantly in the *gat1* seedlings compared to the wild type under FN, low C, low N conditions respectively (Table 1). Furthermore, comparing the levels of metabolites of plants grown in the different media showed that the vast majority of the significantly changed metabolites are common to two or all three of the tested media (Figure 1).

**Table 1:** **Metabolites that level changed significantly in the *gat1* samples compared to *ws* under the tested conditions according to the Student's *t*-test (*n* = 4) with confidence interval 95%**.

**FN medium**	***t-*value**	**Low C medium**	***t*-value**	**Low N medium**	***t*-value**
Glu	3.4	Glu	3.5	Glu	4.4
Asn	4.3	Asn	8.8	Asn	11.0
Met	4.0	Lys	3.7	Lys	9.6
Ala	5.2	Met	2.7	Ala	6.5
Ornithine	6.6	Ala	5.6	β-Ala	6.8
Gln	4.3	β-Ala	6.0	Ornithine	6.8
Tyr	2.6	Gly	6.0	Gly	10.0
Arg	6.9	Tyr	4.0	Gln	24.3
Maltose	9.0	Arg	11.2	Tyr	5.7
Malate	27.9	Sucrose	17.9	Arg	10.9
Malic acid-2-methyl	6.3	Maltose	5.5	Maltose	14.3
Fumarate	10.2	Glycerol	22.6	Glycerol	12.6
Citrate	15.4	Inositol	8.0	Malate	2.9
Glyceric acid	5.9	Malate	14.1	Fumarate	16.6
Succinate	22.2	Malic acid-2-methyl	7.4	Glyceric acid	9.8
Dehydroascorbic acid	5.9	Fumarate	11.0	Succinate	3.1
Melibiose	4.5	Citrate	22.6	Dehydroascorbic acid	17.0
Spermine	2.6	Glyceric-acid	4.1	Lactate	4.7
Adenosine-5-MP	9.3	Succinate	7.2	Adenine	5.8
Benzoic acid	6.2	Dehydroascorbic acid	7.0	GABA	18.1
Nicotinic acid	5.9	GABA	5.2	Spermine	5.1
Rhamnose	10.6	Adenosine-5-MP	3.1	Adenosine -5-MP	17.0
Fructose	3.4	Benzoic acid	4.4	Benzoic acid	4.9
Glucaric acid-1,4-lactone	4.2	Nicotinic acid	4.1	Glucaric acid-1,4-lactone	16.7
Turanose	9.0	Glucose-6P	3.6	Rhamnose	13
Palatinose	4.5	Fructose	3.3	Turanose	4.9
		Glucose	3.7	Kestose	2.9
				Glucose	11.1

**Figure 2:**
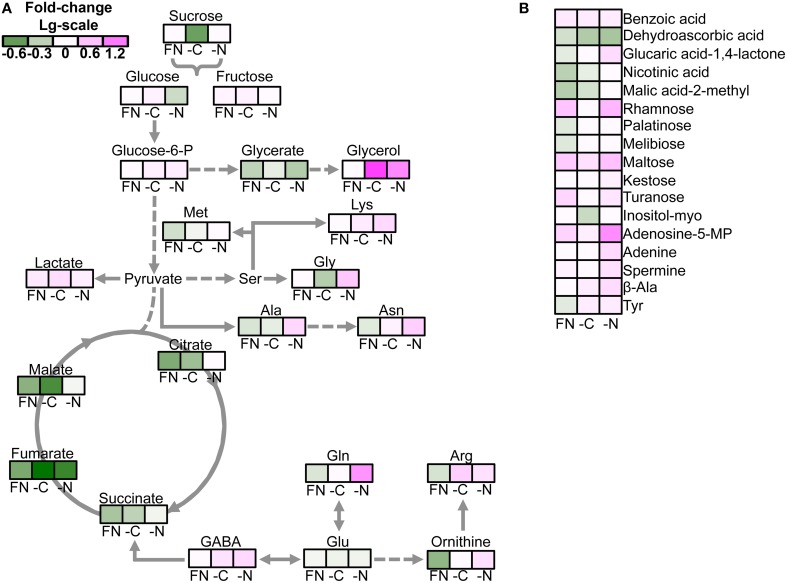
**Effect of media on the metabolite profile of *gat1* compared to *ws***. The false color heat-map of the fold-changes in the levels of metabolite between *gat1* and *ws* under FN, low C, and N media represented in the view of major metabolic pathways **(A)** and the rest of metabolites **(B)**. Only significant changes between the genotypes according to the Student's *t*-test with confidence interval 95% are presented. Data are fold-change of means (*n* = 4) in Log_10_-scale.

The quantitative analysis of the differences between *gat1* and *ws* revealed a common pattern of change for the majority of the carboxylic acids and sugars across plants grown in all tested media. The abundance of most of these metabolites was lower in *gat1* compared to *ws* with exception of lactate, benzoate, maltose, and rhamnose, which accumulated significantly in the mutant (Figure 2). By contrast, the effect of GAT1 deficiency on amino acid metabolism was dependent on the media. Specifically the patterns of change in AA content differ under low N compared to FN and low C (Figure 2). A significant decrease was detected in *gat1* compared to *ws* for methionine, glutamine, asparagine, arginine, ornithine under FN, glycine under low C, and alanine under both media. By contrast, a significant increase (from weak of methionine level to very strong of glutamine level) of these AAs was measured under low N. Unexpectedly, increased levels of GABA were measured in *gat1* under and C and N- deficient media compared to *ws*, while no changes in the non-protein amino acid were detected between the two genotypes under FN (Table 1, Figure 2).

### The opposite response of amino acid metabolism and the metabolism of carboxylic acids and sugars to C and N limitation was detected for both *gat1* and *ws* seedlings

When compared to *ws* (Batushansky et al., 2014), the *gat1* genotype showed similar trends in the metabolite response to medium compositions. Compared to FN medium, low N affected specifically the metabolism of amino acids (Supplementary Figure 2). The level of most of amino acids of both genotypes decreased under low N in comparison to the FN medium. Specifically, the level of alanine, arginine, asparagine, glutamine and ornithine was reduced to 4–8 times as compared to FN medium. Nevertheless, the magnitude of decrease differed between the genotypes (Figure 2), namely *gat1* maintained a higher level of amino acids than *ws* under low N. Unexpectedly, the aromatic amino acids (AAA) tyrosine, phenylalanine kept a similar level under C and N deficiency (Supplementary Figure 1). Relatedly, the AAA-precursor shikimate was measured at comparable levels under low N and FN (Supplementary Figure 2).

Low C induced changes in the content of TCA cycle intermediates and sugars (Supplementary Figures 2, 3). Specifically the levels of malate, citrate, succinate, and lactate in low C medium were 25 and 12.5% lower than in FN medium (Supplementary Figure 2); the levels of the primary sugars sucrose, glucose, and fructose decreased to 25% their level in FN (Supplementary Figure 3). These changes were very close to those in *ws* (Batushansky et al., 2014), demonstrating a general similarity of the response to different medium composition of the mutant and WT. It should be noted that low C also affected the content of some of amino acids. However, in contrast to the effect on the levels of carboxylic acids and sugars, the effect on amino acids content was only moderate (Supplementary Figure 1). Exceptionally among the amino acids, under low C serine and ornithine levels increased 1.5–3 times in comparison with the FN medium (Supplementary Figure 1).

### The global effect of exogenous GABA on seedling metabolism under C- and N-limiting conditions

**Figure 3:**
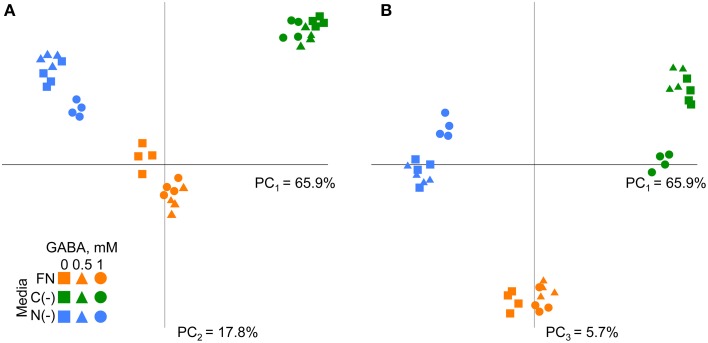
**Principal component analysis of metabolic profiles of *Arabidopsis gat1* genotype under different conditions**. **(A)** First principal component (PC_1_) and second principal component (PC_2_) are plotted on the axes. **(B)** First principal component (PC_1_) and third principal component (PC_3_) are plotted on the axes. Variance explained by each component is indicated on the plot.

The metabolic dataset was next analyzed by principle component analysis (PCA). The first three PCs explained more than 89% of total variance within the metabolite dataset among samples (Figure 3). The distribution of the samples across the 1st principal component (Figure 3A), which accounted for 65.9% of total variance, generates distinct groups according to the plant growing media and reflects the underlying differences in metabolism similarly to WT (Supplementary Figure 4). By extracting the eigenvalues we identified those metabolites that mostly affected the separation between samples on the 1st, 2nd, and 3rd components (Supplementary Table 1). Interestingly, glutamine was one of the most affected metabolites accordingly to first two components, demonstrating its high sensitivity to media composition. Together with glutamine other N-rich compounds asparagine, arginine, ornithine, and spermidine contributed to the high variability among the samples under different conditions (Supplementary Table 1). Alanine, glycine, lysine, saccharic acids, glucose, threonine, glycerol, and proline had the strongest contribution to the distribution of the samples across 3rd component accounting less than 6% of total variance (Figure 3B, Supplementary Table 1). Metabolic differences were identified between the samples supplemented with GABA (1 mM) from untreated samples and those supplemented with GABA 0.5 mM under nutrient-limited conditions (see dispersion of samples on PC_2_ and PC_3_ in Figures 3A,B). Under FN separation was observed between GABA treated samples (either 0.5 or 1 mM) and non-treated (Figure 3). To statistically validate the implications derived from the PCA, the dataset was subjected to One-Way analysis of variance (ANOVA), with confidence interval 95% and Bonferroni correction. The effect of GABA on metabolism was visibly less specific to the culture medium as compared to the results obtained for the wild type (Batushansky et al., 2014). The effect of different concentrations of exogenous GABA on the metabolites level was significant for nine compounds (20%) under FN and low C media and for four compounds under low N medium (Supplementary Table 2).

**Figure 4:**
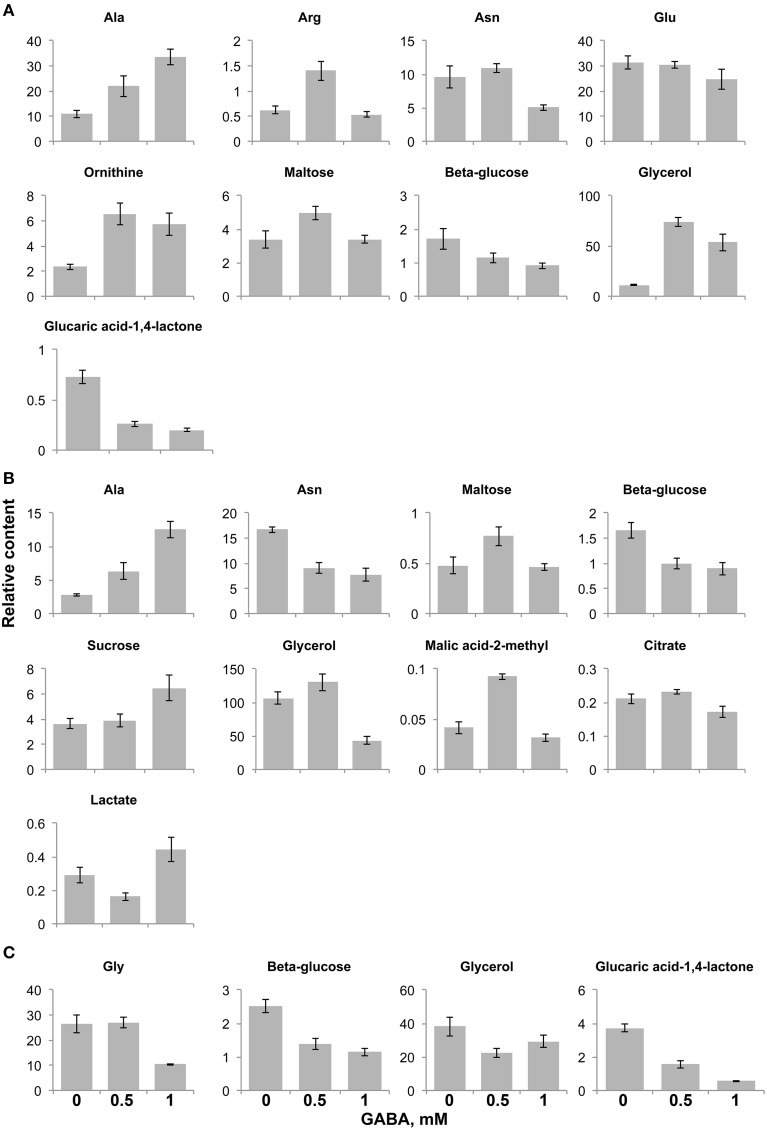
**Significantly changed metabolite content in the *gat1* samples in respect to increasing GABA treatment under FN- (A), low C- (B), and low N-media (C)**. Bar graphs represent a relative content of metabolites (Y axis) after normalization by fresh weight and standard ribitol. Data are mean (*n* = 4) ± SD. Statistical significance was tested with One-Way ANOVA, *p*_BONFERRONI_ ≤ 0.05.

Endogenous GABA was not affected in content by the addition of exogenous GABA in the mutants, but it increased in the *ws* (Batushansky et al., 2014) demonstrating the role of GAT1 in GABA influx into the cell. The level of asparagine significantly dropped in content (to 50%) under both FN and low C media with supplemental GABA (Figure 4). The level of alanine also changed consistently under both media in response to GABA, increasing in a range from 2–3 fold (0.5 mM GABA) to 3–4 times (1 mM GABA). GABA treated seedlings strongly accumulated glycerol (4–6 fold increased) under FN (Figure 4A) while it decreased under low C medium by 50% (Figure 4B) and under low N (Figure 4C). Citrate and lactate significantly changed under low C, but if citrate slightly decreased in presence of 1 mM GABA, the level of lactate increased 1.5-fold under the same conditions (Figure 4B). The samples under low N supplied with 1 mM GABA showed strong decrease of glycine (Figure 4C). On the contrary, the changes in the level of the GABA precursor glutamate were detected only under FN medium supplied with 1 mM GABA where glutamate slightly, but unexpectedly, decreased (Figure 4A). Last, a drop in the relative content of β-glucose under all tested media supplied with both GABA concentrations was observed (Figure 4, Supplementary Table 2). Taken together the GABA dependent metabolic response in contrast to previously published results on *ws* (Batushansky et al., 2014) was mostly irrespective of biochemical class of the affected metabolites and medium compositions.

### Whole-genome transcript analysis of *gat1* and exogenous GABA

**Figure 5:**
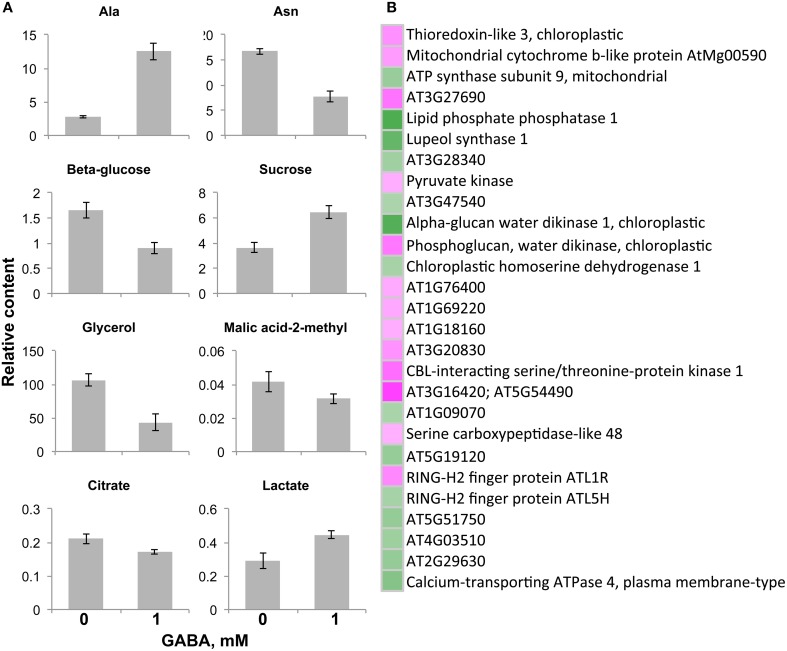
**Significantly changed metabolite content and gene expression levels in the *gat1* samples in respect to 1 mM GABA treatment under low C medium**. Bar graphs **(A)** represent a relative content of metabolites (Y axis) after normalization by fresh weight and standard ribitol. Data are mean (*n* = 4) ± SD. Statistical significance was tested with Student's *t*-test, *p*_BONFERRONI_ ≤ 0.05. False color heat-map **(B)** represents fold-changes in the levels of metabolic genes expression between 1 mM GABA-treated samples and untreated samples under low C medium. Only significantly changed (*p*_FDR_ ≤ 0.05, 4 times fold-change) and functionally annotated metabolic genes are presented.

**Figure 6:**
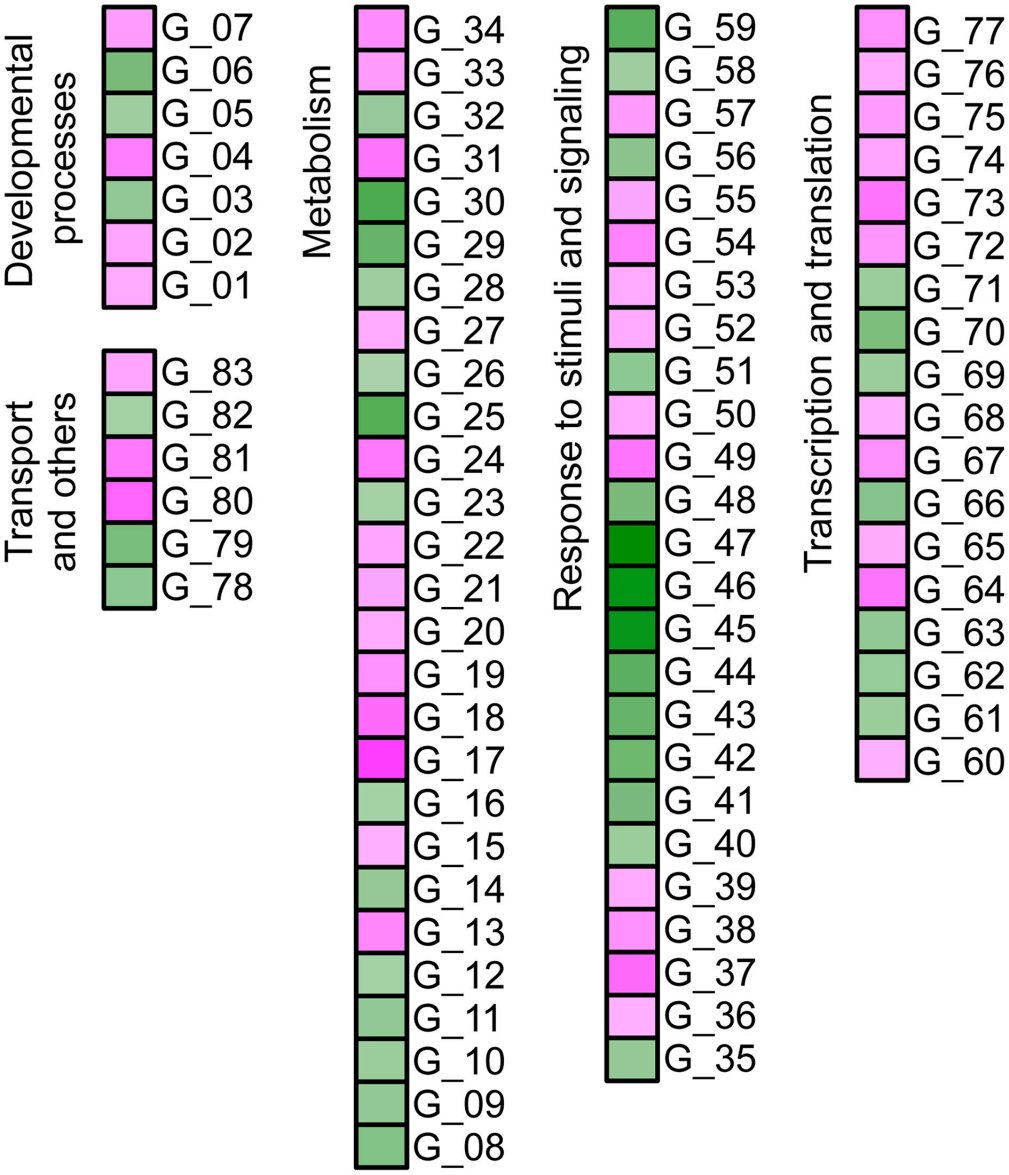
**Effect of 1 mM exogenous GABA on gene expression profile of *gat1* samples under low C conditions**. Only significantly changed (*p*_FDR_ ≤ 0.05, 4 times fold-change) and functionally annotated genes are presented (see Supplementary Data 5 for details).

Considering the broadly similar effect of medium composition on metabolic classes between *gat1* and *ws*, gene expression of both genotypes was compared under FN conditions only. The results, when filtered as described in Materials and methods, showed a significant change in expression of 796 genes (393 up- and 403 down-regulated) in *gat1* plants compared to *ws* (Supplementary Data 1). Following DAVID enrichment analysis and taking into consideration only an enrichment score higher than 2 (Huang da et al., 2009) five clusters for up-regulated and six clusters for down-regulated genes were selected based on the GOTERM database (Supplementary Data 2). These results revealed that up-regulated genes in *gat1* were mainly involved in the response to abiotic stimuli such as heat and light. However, more interestingly, we additionally identified up-regulation of CaM-binding transcription activator 1 (At5g09410) and glutamate dehydrogenase 2 (At5g07440), both of which are involved in GABA metabolism. The functional annotation of down-regulated genes showed that most of them were involved in cell wall metabolism (group of peroxidases), transport systems (aquaporin), and chloroplastic activity. Additionally, a small group of genes involved in phenylpropanoid pathway was detected, namely aspartate aminotransferase (At2g30970), chorismate mutase (At3g29200) and cytochrome P450 (At2g34770). We additionally tested the effect of 1 mM exogenous GABA on gene expression levels in *gat1* under low C deficiency. Results from this experiment revealed a significant difference in expression of 136 genes (62 up- and 74 down-regulated). Following functional annotation we identified that the up-regulated genes in the samples treated with exogenous GABA mostly belonged to chloroplastic activity and abiotic stimulus gene classes (Figures 5, 6, Supplementary Data 3), whilst down-regulated genes were involved in the response to endogenous stimulus, mostly ethylene-responsive transcriptional factors (At1g28370, At3g20310, At1g77640); (Figure 5, Supplementary Data 4).

## Discussion

Several amino acids permeases have been isolated in *Arabidopsis*, whose main substrates were glutamate, glutamine and aspartate, with low or no affinity to GABA (Fischer et al., 2002; Meyer et al., 2006). The first plant transporter with high affinity to GABA was identified by Meyer et al. (2006). Despite the authors did not demonstrate a direct flux of GABA via GAT1 protein in plant cell, its role in GABA transport was shown in yeast by complementation strategy. Moreover, *gat1* expression was detected in plant tissues particularly under conditions associated with wounding or senescence and it was in correlation with GABA concentration (Meyer et al., 2006). In spite of an increasing number of studies suggesting a pivotal role of GABA in C-N metabolism, the functional significance of its transport in respect to central cellular metabolism has yet to be addressed. Here the metabolism of the plasma membrane localized GABA transporter deficient mutant was investigated under FN and nutrient deficient media. Changes caused by both C- and N-deficiency in comparison with FN medium in *gat1* were similar in trend but significantly sharper in magnitude when compared to those in *ws* (Figure 2), suggesting a significant role of the GABA transporter under C/N deficiency in the seedling metabolism. Our results find support in one of the few research exploring GABA effect on plant metabolism: in *Brassica napus* GABA treatment induced significant changes in the expression of BnNrt2, suggesting that it could act as a putative long-distance inter-organ signal molecule in plants in respect to N metabolism (Beuve et al., 2004).

Generally, C- and N-limiting conditions resulted in decreased content of most metabolites in comparison with FN medium, but the degree of this decline varied. Low N induced an expected reduction in the content of most amino acids. These data support previous works. For example Urbanczyk-Wochniak and Fernie showed a wide impact of nitrate deficiency on metabolism, whose main effect was a decrease in the level of amino and carboxylic acids (Scheible et al., 2004; Urbanczyk-Wochniak and Fernie, 2005). The effect of low N on glutamine and asparagine—AA with the central role in N assimilation, storage, and transport within the plant (Coruzzi and Bush, 2001)—in *gat1* was even greater compared to *ws*. Specifically under low N *gat1* showed higher content for asparagine, glutamine, arginine, lysine, ornithine compared to *ws*. The endogenous content of GABA in *gat1* was also significantly higher than that in *ws* under low N and low C conditions and at comparable level under FN medium (Figure 2). In striking contrast, the content of glutamate, while maintaining relatively high (10 time higher than glutamine) in both *gat1* and *ws*, was lower in the mutant under all media. The different response of the closely related glutamate and glutamine to the medium conditions can be thus attributed to glutamate central position in amino acid metabolism (Stitt et al., 2002) or to the reversibility of amino acid transferase reactions as suggested by Forde and Lea (2007). Taken together, these metabolic data could suggest that the glutamate conversion to GABA is shifted toward the production of the other amino acids. A limited export of GABA could lead to its accumulation in the cell; enhanced transamination reactions of GABA would follow and contribute to other amino acids. Furthermore GABA-induced feedback inhibition of glutamate decarboxylase (Porter and Martin, 1984) would eventually lead to glutamate repartitioning to different amino acids.

In contrast to a decreased amino acid pool under N limitation we measured an accumulation of malate in both genotypes compared to FN. Malate accumulation under N deficiency was already observed (Pasqualini et al., 2001) and it could be attributed to a transient storage of C (Recht et al., 2014). However, when comparing *gat1* to *ws*, a higher GABA to glutamate ratio (in the first) was accompanied by lower abundance of virtually all TCA cycle intermediates, namely succinate, malate, citrate, and fumarate. The up-regulation of glutamate dehydrogenase (GDH) in the mutant and increased pool of free amino acids likely supports the hypothesis of a repartitioning of 2OG to glutamate-derived amino acids, emphasizing the link between GABA-glutamate metabolism and GDH, “one of the key checkpoints controlling the C-N status of the plant” (Tercé-Laforgue et al., 2004). Under the proposed scenario of a limited GABA efflux inducing glutamate-derived amino acids biosynthesis and cataplerotic TCA cycle activity, *gat1* is suggested to modulate C-N metabolism. A very recent report found that GABA modulates plant growth by directly regulating the activity of another transporter, a plant-specific anion transporters for malate (Ramesh et al., 2015). Accumulation of GABA decreased malate efflux by direct binding to the ALMT transporter. This is the first report showing a direct effect of GABA as a signaling molecule mediating cellular metabolism, ending more than a decade-long debate. In our study, malate did not accumulate in the mutant under any media in spite of GABA endogenous accumulation. However, when exogenous GABA was added to the medium, in the WT genotype enhanced influx of GABA led to its accumulation under low C conditions, and it was accompanied with further decrease in malate and citrate content (compared to FN). In the *gat1* mutant, GABA feeding caused no changes in GABA content under low C, very likely due to limited GABA influx. The absence of the one specific GABA transporter can be partly compensated by other quaternary transporters (i.e., two known amino acid transporters, amino acid permease 3, AAP3, and the proline transporters 2, ProT2, potentially transport GABA). However, in plants like in animals and bacteria, related compounds like proline compete with GABA and inhibit its transport via—for instance—ProT2. Supplemented GABA to *gat1* seedlings led to a significant and unexpected alteration in β-glucose and glycerol (lines of evidence in mammals suggest GABA inhibits glucagon secretion and glucose in blood, (Bailey et al., 2007). GABA treatment in *gat1* mutant also significantly affected the regulation of pyruvate kinase, an enzyme involved in the production of pyruvate from PEP, that is the entry point between the glycolysis and the TCA cycle. Furthermore, we detected a decrease of malic acid-2-methyl, substrate for pyruvate production, and citrate following 1 mM GABA feeding (Figure 5). Thus a role of GABA in the regulation of C metabolism is very likely and suggested by (i) previous studies in transgenic *Arabidopsis* plants with a deregulated GAD which showed alterations in N to C ratio in *Arabidopsis* seeds (Fait et al., 2011) and by (ii) the effect of GABA on the metabolism of *ws Arabidopsis* seedlings under C deficiency (Batushansky et al., 2014) or when grown in darkness (Araújo et al., 2010). Having said that, surprisingly the malate content in the mutant under low C did not change when exogenous GABA was applied. Under the very recent scenario proposed by Ramesh, we would suggest that increased extracellular GABA (by the feeding treatment) bound to the ALMT site, which by convention are extracellular preventing malate efflux. It should be noted that this is a hypothesis prompted by the very recent findings by Ramesh (Ramesh et al., 2015). While tempting, in our study we did not distinguish between intra and extracellular partitioning of metabolites and the ALMT binding site location needs validation. Future studies will need to test the validity of this hypothesis.

Last, among the 393 up-regulated genes in the mutant a few are involved in phenylpropanoid metabolism, including chorismate mutase of the shikimate pathway. The link between GABA and secondary metabolism is unclear, however a targeted enhancement of glutamate decarboxylase in *Arabidopsis* seeds was previously shown to lead to the up-regulation of genes of the shikimate pathway and tryptophan metabolism (Fait et al., 2011). Future studies should try to address the link between the GABA shunt and the shikimate metabolism.

To conclude, the present study on the effects of GABA feeding in different media on *Arabidopsis gat1* mutant seedlings suggest a GABA mediated regulation of C to N metabolism possibly by the newly discovered ALMT. Further studies are needed to dissect between the metabolic and signaling role of GABA in mediating changes in cellular metabolism.

## Conflict of interest statement

The authors declare that the research was conducted in the absence of any commercial or financial relationships that could be construed as a potential conflict of interest.
