# Supplementary material for: The transporter GAT1 plays an important role in GABA-mediated carbon-nitrogen interactions in Arabidopsis
Source: Front Plant Sci. 2015 Sep 29;6:785. doi: 10.3389/fpls.2015.00785 (PMC4586413; doi:10.3389/fpls.2015.00785)
Supplement: Supplementary file 1 [file Table1.DOCX]

***Supplementary Material***

The Transporter GAT1 Plays an Important Role in GABA-mediated Carbon-Nitrogen Interactions in *Arabidopsis*

Albert Batushansky^1^, Menny Kirma^2^, Nicole Grillich^3^, Phuong Anh Pham^3^, Doris Rentsch^4^, Gad Galili^2^, Alisdair R Fernie^3^, and Aaron Fait^1*^

^1^The Jacob Blaustein Institutes for Desert Research, Ben-Gurion University of the Negev, Midreshet Ben-Gurion, Israel, ^2^Department of Plant Science, Weizmann Institute of Science, Rehovot, Israel, ^3^Max-Planck Institute of Molecular Plant Physiology, Potsdam-Golm, Germany, ^4^Institute of Plant Sciences, University of Bern, Bern, Switzerland

^*^Correspondence: Prof. Aaron Fait, The Ben-Gurion University of the Negev, The French Associates Institute for Agriculture and Biotechnology of D,rylands, The Jacob Blaustein Institutes for Desert Research, Laboratory of Plant metabolism, Midreshet Ben-Gurion, 84990, Israel, E-mail: [fait@bgu.ac.il](mailto:fait@bgu.ac.il)

**Supplementary table 1. Eigenvectors values (in descending order) of metabolites were calculated by PCA algorithm for 1^st^ and 3^rd^ components of *gat1* genotype**

| Name | PC_1_ | Name | PC_2_ | Name | PC3 |
| --- | --- | --- | --- | --- | --- |
| Glucaric acid-1,4-lactone | 2.1 | Gln | 4.1 | Ala | 4.2 |
| Arg | 2.1 | Succinate | 2.6 | Gly | 3.6 |
| Gln | 2.1 | Kestose | 2.2 | Lys | 1.6 |
| Ornithine | 2.1 | Raffinose | 2.2 | Glucaric acid-1,4-lactone | 1.5 |
| Succinate | 1.9 | Maltose | 2.0 |  |  |
| Raffinose | 1.7 | Asn | 1.8 |  |  |
| Kestose | 1.7 | Lactate | 1.7 |  |  |
| Malate | 1.7 | Sucrose | 1.6 |  |  |
| Lactate | 1.5 | Malic acid-2-methyl | 1.6 |  |  |
| Glycerate | 1.5 |  |  |  |  |
