# Supplementary material for: The transporter GAT1 plays an important role in GABA-mediated carbon-nitrogen interactions in Arabidopsis
Source: Front Plant Sci. 2015 Sep 29;6:785. doi: 10.3389/fpls.2015.00785 (PMC4586413; doi:10.3389/fpls.2015.00785)
Supplement: Supplementary file 2 [file Table2.DOCX]

***Supplementary Material***

The Transporter GAT1 Plays an Important Role in GABA-mediated Carbon-Nitrogen Interactions in *Arabidopsis*

Albert Batushansky^1^, Menny Kirma^2^, Nicole Grillich^3^, Phuong Anh Pham^3^, Doris Rentsch^4^, Gad Galili^2^, Alisdair R Fernie^3^, and Aaron Fait^1*^

^1^The Jacob Blaustein Institutes for Desert Research, Ben-Gurion University of the Negev, Midreshet Ben-Gurion, Israel, ^2^Department of Plant Science, Weizmann Institute of Science, Rehovot, Israel, ^3^Max-Planck Institute of Molecular Plant Physiology, Potsdam-Golm, Germany, ^4^Institute of Plant Sciences, University of Bern, Bern, Switzerland

^*^Correspondence: Prof. Aaron Fait, The Ben-Gurion University of the Negev, The French Associates Institute for Agriculture and Biotechnology of D,rylands, The Jacob Blaustein Institutes for Desert Research, Laboratory of Plant metabolism, Midreshet Ben-Gurion, 84990, Israel, E-mail: [fait@bgu.ac.il](mailto:fait@bgu.ac.il)

**Supplementary table 2. Results of one-way ANOVA of *gat1* genotype. GABA concentration was used as a condition for analysis. Values of metabolites shown in the table are significantly different at *p*=0.05 with Bonferroni correction.**

| FN | Low C | Low N |
| --- | --- | --- |
| Asn | Asn | Gly |
| Ala | Ala | Glycerol |
| Ornithine | Sucrose | Beta-glucose |
| Glu | Maltose | Glucaric acid-1,4-lactone |
| Arg | Glycerol |  |
| Maltose | Malic acid-2-methyl |  |
| Glycerol | Citrate |  |
| Beta-glucose | Lactate |  |
| Glucaric acid-1,4-lactone | Beta-glucose |  |
