# Supplementary material for: The transporter GAT1 plays an important role in GABA-mediated carbon-nitrogen interactions in Arabidopsis
Source: Front Plant Sci. 2015 Sep 29;6:785. doi: 10.3389/fpls.2015.00785 (PMC4586413; doi:10.3389/fpls.2015.00785)
Supplement: Supplementary file 8 [file Image1.PDF]

Supplementary Material

The Transporter GAT1 Plays an Important Role in GABA-mediated Carbon-Nitrogen Interactions in *Arabidopsis*

Albert Batushansky[1], Menny Kirma[2], Nicole Grillich[3], Phuong Anh Pham[3], Doris Rentsch[4], Gad Galili[2], Alisdair R Fernie[3], and Aaron Fait[1*]

[1]The Jacob Blaustein Institutes for Desert Research, Ben-Gurion University of the Negev, Midreshet Ben-Gurion, Israel, [2]Department of Plant Science, Weizmann Institute of Science, Rehovot, Israel, [3]Max-Planck Institute of Molecular Plant Physiology, Potsdam-Golm, Germany, [4]Institute of Plant Sciences, University of Bern, Bern, Switzerland

[*]Correspondence: Prof. Aaron Fait, The Ben-Gurion University of the Negev, The French Associates Institute for Agriculture and Biotechnology of D,rylands, The Jacob Blaustein Institutes for Desert Research, Laboratory of Plant metabolism, Midreshet Ben-Gurion, 84990, Israel, E-mail: fait@bgu.ac.il

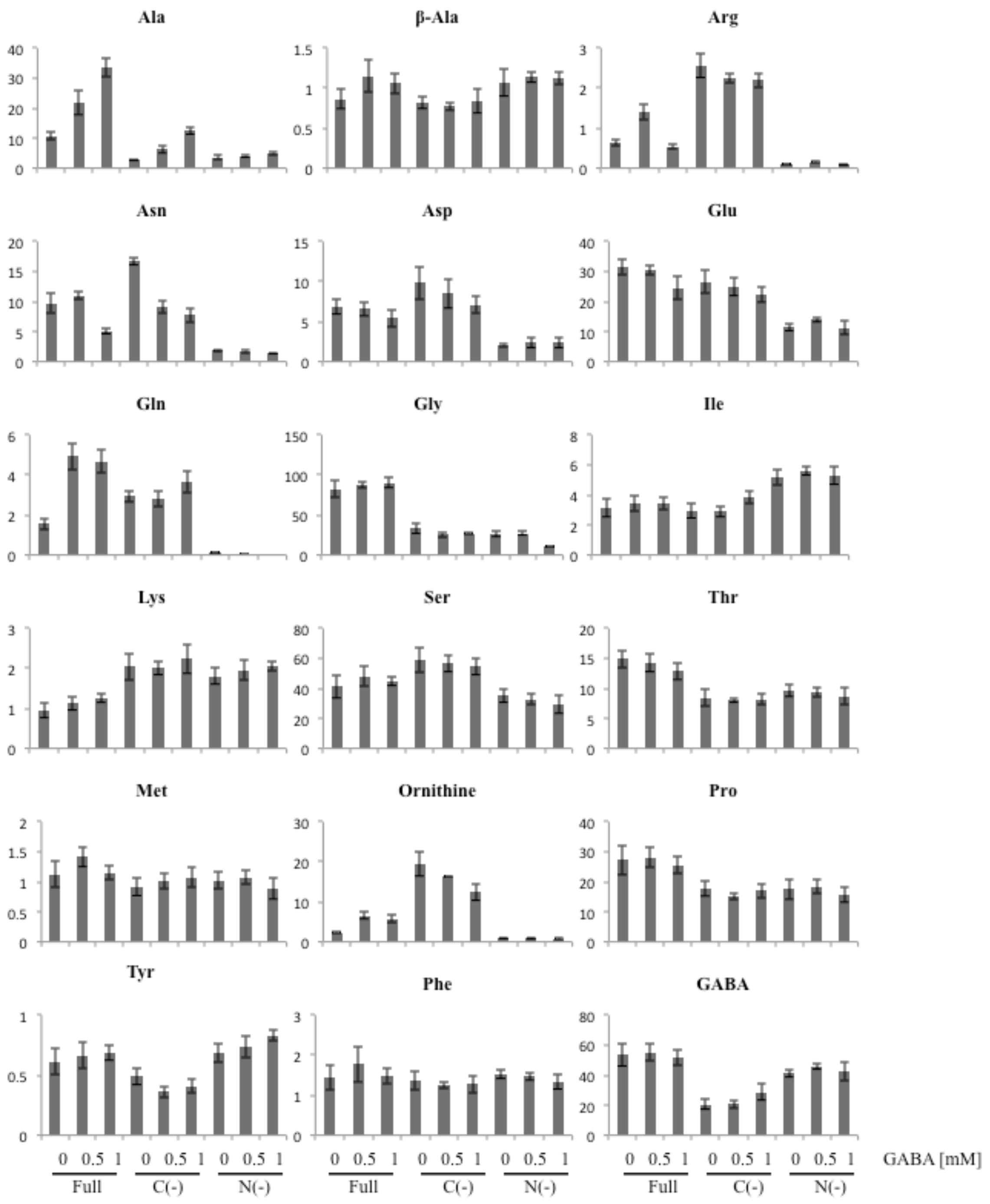

Supplementary figure 1. Changes in amino acids content of in vitro-grown *gat1* genotype in response to different media and GABA treatment. Relative content (Y axis) is given according to normalization by fresh weight and ribitol. Data are mean (n=4) ± SD.
