# Supplementary material for: The transporter GAT1 plays an important role in GABA-mediated carbon-nitrogen interactions in Arabidopsis
Source: Front Plant Sci. 2015 Sep 29;6:785. doi: 10.3389/fpls.2015.00785 (PMC4586413; doi:10.3389/fpls.2015.00785)
Supplement: Supplementary file 11 [file Image4.PDF]

Supplementary Material

The Transporter GAT1 Plays an Important Role in GABA-mediated Carbon-Nitrogen Interactions in *Arabidopsis*

Albert Batushansky[1], Menny Kirma[2], Nicole Grillich[3], Phuong Anh Pham[3], Doris Rentsch[4], Gad Galili[2], Alisdair R Fernie[3], and Aaron Fait[1*]

[1]The Jacob Blaustein Institutes for Desert Research, Ben-Gurion University of the Negev, Midreshet Ben-Gurion, Israel, [2]Department of Plant Science, Weizmann Institute of Science, Rehovot, Israel, [3]Max-Planck Institute of Molecular Plant Physiology, Potsdam-Golm, Germany, [4]Institute of Plant Sciences, University of Bern, Bern, Switzerland

[*]Correspondence: Prof. Aaron Fait, The Ben-Gurion University of the Negev, The French Associates Institute for Agriculture and Biotechnology of Drylands, The Jacob Blaustein Institutes for Desert Research, Laboratory of Plant metabolism, Midreshet Ben-Gurion, 84990, Israel, E-mail: fait@bgu.ac.il

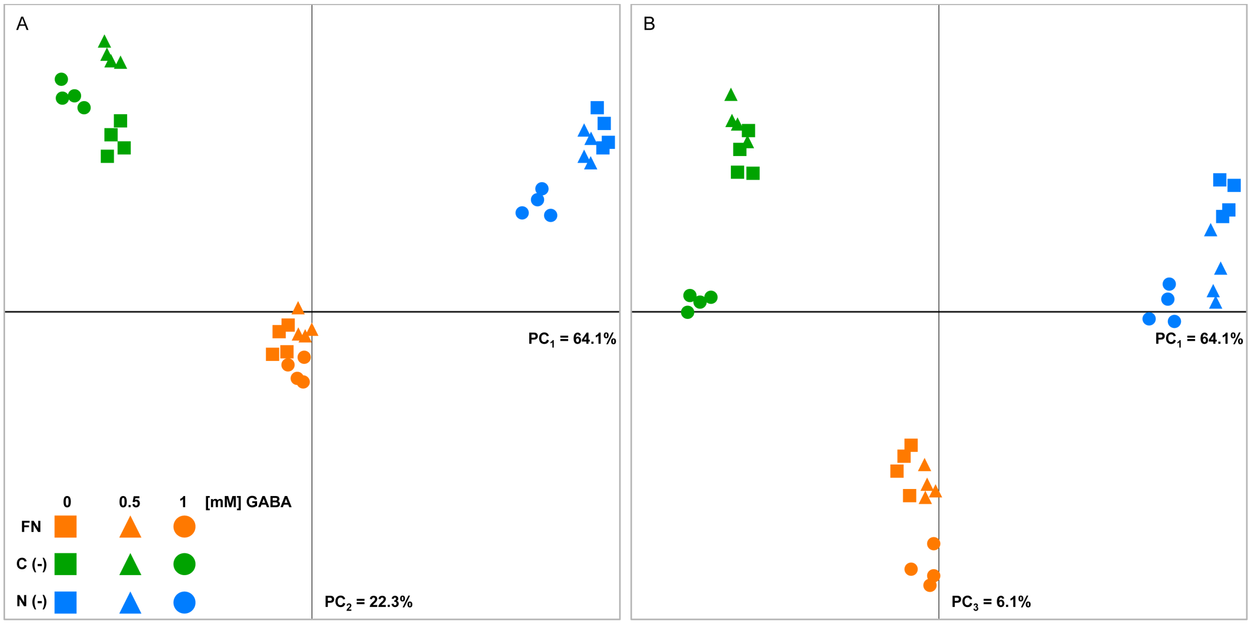

Supplementary figure 4. Principal component analysis of metabolic profiles of *Arabidopsis ws* genotype under different conditions (see legend). A: first principal component (PC_1) and second principal component (PC_2) are plotted on the axes. B: first principal component (PC_1) and third principal component (PC_3) are plotted on the axes. Variance explained by each component is indicated on the plot. The figure was taken from the Batushansky et al., 2014 with the permission of copyrighter Molecular Plant journal.
